# Comparison of rhBMP-2 in Combination with Different Biomaterials for Regeneration in Rat Calvaria Critical-Size Defects

**DOI:** 10.1155/2022/6281641

**Published:** 2022-04-25

**Authors:** Francisca Uribe, Bélgica Vásquez, Juan Pablo Alister, Sergio Olate

**Affiliations:** ^1^Medical Sciences, Faculty of Medicine, University of La Frontera, Temuco, Chile; ^2^Division of Oral, Facial and Maxillofacial Surgery, Faculty of Dentistry, University of La Frontera, Temuco, Chile; ^3^Center of Excellence in Surgical and Morphological Studies (CEMyQ), University of La Frontera, Temuco, Chile; ^4^Faculty of Health Sciences, Universidad de Tarapacá, Arica, Chile

## Abstract

Regeneration of critical bone defects requires the use of biomaterials. The incorporation of osteoinductive agents, such as bone morphogenetic proteins (BMPs), improves bone formation. This study aimed to compare the efficacy of rhBMP-2 in combination with different materials for bone regeneration in critical-sized rat calvarial defects. This was an experimental animal study using 30 rats. In each rat, two 5-mm critical-size defects were made in the calvaria (60 bone defects in total) using a trephine. All rats were randomized to one of the six groups: control (C), autograft + rhBMP-2 (A), absorbable collagen sponge + rhBMP-2 (ACS), *β*-tricalcium phosphate + rhBMP-2 (B-TCP), bovine xenograft + rhBMP-2 (B), and hydroxyapatite + rhBMP-2 (HA). The outcome was assessed after 4 and 8 weeks using histological description and the histological bone healing scale. Statistical analysis was performed using the Kruskal-Wallis and Mann–Whitney *U* tests, with a *p-*value set at 0.05. The average bone healing scores per group were as follows: C group, 12.5; A group, 26.5; ACS group, 18.8; B-TCP group, 26.2; HA group, 20.9; and B group, 20.9. The C group showed a significant difference between weeks 4 and 8 (*p* = 0.032). Among the 4-week groups, the C group showed a significant difference compared to A (*p* = 0.001), ACS (*p* = 0.017), and B-TCP (*p* = 0.005) groups. The 8-week experimental group did not show any significant differences between the groups. The 5-mm critical size defect in rat calvaria requires the use of bone biomaterials to heal at 4 and 8 weeks. rhBMP-2, as applied in this study, showed no difference in new bone formation when combined with bovine, B-TCP, or HA biomaterials.

## 1. Introduction

The size of a bone defect is a key factor in bone regeneration, and in the case of larger defects, defined as critical, it is necessary to include materials to facilitate new bone formation [1, 2]. Autografts continue to be the gold standard because of their osteoconductive, osteoinductive, and osteogenic properties [3]. However, they are associated with donor sites anatomical limits as well as associated morbidity. This warrants development of alternative therapies [4, 5].

Allografts, xenografts, and alloplastic grafts have been frequently used in the maxillofacial region. These materials have proven clinical applications and optimal responses [6, 7]; however, the potential risk of infection, immunogenicity, or rejection of the implanted material cannot be ignored [8–10]. In addition, alloplastic materials do not have osteoinductive or osteogenic properties like autogenous grafts; thus, osteoinductive agents such as proteins, drugs, or growth factors have been incorporated into biomaterials. Bone morphogenetic proteins (BMPs) are an example of such incorporation. They are an endogenous group of proteins that belongs to the transforming growth factor beta (TGF-*β*) family. They act mainly as cytokines that mediate the differentiation of mesenchymal cells into bone- and cartilage-forming cells. In particular, recombinant human bone morphogenetic protein-2 (rhBMP-2) has been identified to play a critical role in bone formation and healing due to its capacity to induce osteoblast differentiation [11, 12].

The use of rhBMP-2 with an absorbable collagen sponge (ACS) matrix, which is currently approved by the FDA, has some disadvantages. The precise positioning of the ACS can be complicated in some types of defects, and in some cases, postoperative displacement has been reported [13]. However, it produces a fast initial release of rhBMP-2 and rapid elimination compared to bone formation [14–16]. Therefore, supraphysiological doses are used to obtain good results and generate bone tissue [17], which creates potential complications, such as edema, erythema, pain, or even infection [18]. In this context, new technologies, models of application, and vehicles are needed to improve performance in terms of effectiveness and biosafety.

Studies have shown differences in outcomes of various vehicles and biomaterials to deliver rhBMP-2. A systematic review by Motamedian et al. [19] found that the most frequently used scaffold was polycaprolactone (PCL), followed by tricalcium phosphate (*β*-TCP), poly-lactic-glycolic acid (PLGA), and material of bovine origin (Bio-Oss). Typically, a scaffold must be biocompatible and present a porosity that is suitable for the infiltration and proliferation of cells and blood vessels produced at the site of new bone formation; in addition, it must be predictably biodegradable, stress- and compression-resistant, sterilizable, and easily handled [20–23].

This study aimed to compare the efficacy of rhBMP-2 in combination with different materials for bone regeneration in critical-sized rat calvarial defects.

## 2. Materials and Methods

An animal experimental study was conducted with 30 Sprague Dawley rats, which were 16 weeks old, healthy, male, and sexually mature, with an average weight of 438 g. Only male rats were used to minimize bias from hormonal effects on bone formation. The rats were kept under standard conditions of humidity, temperature, and light, with two rats per cage, and fed commercial pellets and water ad libitum throughout the experiment. All animal experimental procedures were performed according to the recommendations of the Guide for the Care and Use of Laboratory Animals [24]. The research project was approved by the Institutional Review Board (IRB) of Universidad de La Frontera (File no. 041/19).

The determination of sample size was based on the bioethical principles of Russell and Burch's 3 Rs (Russell and Burch, 1959) for animal experimentation: replacement, reduction, and refinement, using the minimum number needed to obtain a significant difference [25].

*Escherichia coli*-derived RhBMP-2 was used (Novosis Daewoong, Korea, 0.25 mg). The rhBMP-2 dose used in each defect was 5 *μ*g, and 0.25 mg rhBMP-2 was dissolved in a 2.5 ml saline solution. Therefore, 50 doses of 50 *μ*l were obtained, each containing 5 *μ*g of rh-BMP-2. Next, 50 *μ*l of this solution was inserted into the biomaterial using a micropipette 10 min before use, as indicated by the manufacturer. The mixture of the biomaterial with the rhBMP-2 is obtained using a specific weight to standardize the proportion of the mixture (0,1 g biomaterial/5 *μ*g rhBMP-2).

**Figure 1:**
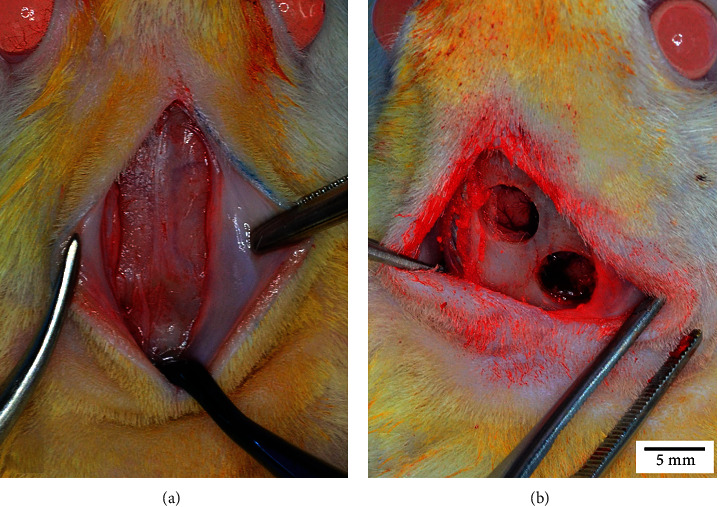
Bone defect in Sprague Dawley rat calvarial. The bone is exposed by incision and retraction of the skin and periosteum covering the calvaria (a). A 5-mm diameter trephine is used to cut the calvarial bone, resulting in two bicortical bone defects (b).

Animals were anesthetized with ketamine 80 mg/kg and xylazine (10 mg/kg). The skull was shaved and scrubbed with an iodine solution. Using sterile instruments and aseptic techniques, a 2% lidocaine + epinephrine 1 : 100,000 dilution to 0.5% infiltration was performed. A cranial skin incision was made in the sagittal midline from the frontal to the occipital bone. A full-thickness flap was lifted to expose the calvaria. Two bicortical bone defects were created using a 5-mm external diameter trephine under continuous irrigation with saline solution. Hemostasis was verified, and the dura mater was preserved (Figure 1).

After this procedure, the rats were randomized to six experimental groups by simple randomization. Group distribution was blinded until the end of the study. The participants were divided into the following groups:

*C group:* control group filled only with blood clot

*A group:* autograft + rhBMP-2; bone defect filled with particulate bone graft removed from the calvaria in the creation of the bone defect

*ACS group:* absorbable collagen sponge + rhBMP-2, filled with Hemospon (Technew, Brazil). A unit of Hemospon (1 cm^2^ cube) was added to each critical size defect. Hemospon is a highly porous lyophilized preparation from purified porcine skin collagen and purified water. It can absorb approximately 45% of its own weight. It is completely reabsorbed in 15 days

*B-TCP group: β*-tricalcium phosphate + rhBMP-2, filled with Excelos (BioAlpha Inc., Korea)

*B group:* bovine xenograft + rhBMP-2, filled with Bio-Oss (Geistlich, Switzerland, 0.25-1 mm)

*HA group:* hydroxyapatite + rhBMP-2, treated with particles of 0.25-1 mm in a nonregular shape. 100% synthetic hydroxyapatite, pure phase greater than 95%, interconnected porosity of 80%, IngeniOs HA (Zimmer-Biomet Inc., USA)

**Figure 2:**
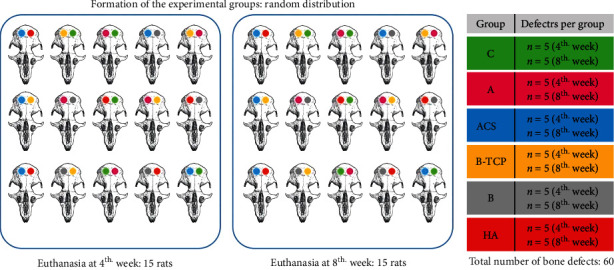
Experimental flow chart. Operating plan and group allocation of this study.

After surgery, the periosteum and muscle were repositioned and sutured using vicryl 4-0, and finally the skin was sutured using nylon 4-0 (Figure 2).

The first 15 rats were euthanized at 4 weeks by an overdose of 160/20 mg/kg ketamine/xylazine (Canadian Council on Animal Care, 1998), and the other 15 rats were euthanized at 8 weeks. The samples were obtained by osteotomies performed with a piezoelectric system (Piezotome, Acteon Satelec, France), fixed in 10% buffered formalin (pH 7.4) for 48 h, demineralized in 10% EDTA buffer for 90 days, subjected to conventional histological processing, and embedded in paraffin (Histosec, Merck). Serial sections 5*-μ*m-thick slices were cut coronally from the center of the bone defect with a microtome (Microm HM 325, Thermo Fisher Scientific Inc., Waltham, MA, USA). The histological slides were stained with hematoxylin and eosin for histological analysis using light microscopy (Leica DM750).

A certified histologist who was blinded to the group distribution performed the histological analysis using a histological bone-healing scale [26]. This scale provides a score for 17 histological parameters (Table 1). The addition of these scores provided the final histological healing score for each defect.

All samples were checked for normal or nonnormal distribution using the Kolmogorov-Smirnov test. To assess whether there was a significant difference between the groups at 4 and 8 weeks, the Kruskal-Wallis test of independent samples was performed with Tamhane's post hoc test. The Mann–Whitney *U* test was used to evaluate differences between 4 and 8 weeks for each group, considering a *p*-value <0.05 for statistical significance.

## 3. Results

### 3.1. Description of Rat Calvarial Bone Tissue

**Figure 3:**
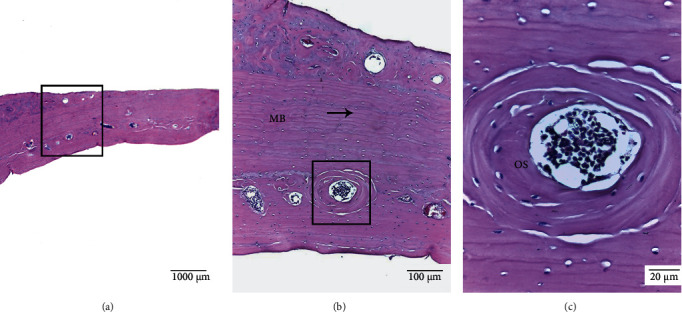
Sprague Dawley rat calvarial bone tissue. (a). Mature compact bone tissue. (b). Mature bone (MB) shows bone lamellae (→) arranged parallel to the surface; in the middle zone, osteons (OS) can be seen; the central canal allows the presence of blood vessels. (c). Osteon (OS) is shown at increased magnification. HE staining.

The bone tissue was formed mainly of compact bone covered by a periosteal membrane of connective tissue containing blood vessels that break through the bone tissue, ensuring bone vascularization. The compact bone tissue was formed by bone lamellae arranged parallel to the surface with multiple osteocytes located in their lagoons. Osteons were observed in the middle zone, with wide-diameter central canals to enable vascularization of the zone (Figure 3).

### 3.2. Histological Characterization of the Experimental Groups

The characterization of the experimental groups using the 17 histological parameters at 4 and 8 weeks after surgery is described in Table 2.

#### 3.2.1. C Group

**Figure 4:**
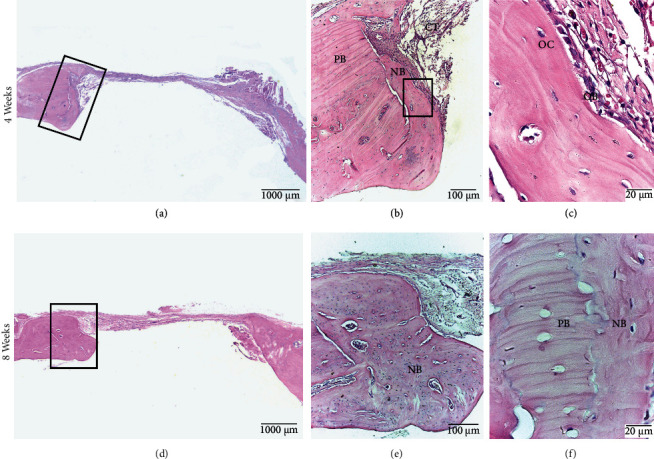
C Group. Calvarial bone defect in male Sprague Dawley rats at 4 weeks (a, b, c) and at 8 weeks (d, e, f). (a) Defect without formation of bone bridge. (b) On the periphery of the defect, the preexisting bone (PB), newly formed immature bone (NB), and connective tissue (CT) can be seen. (c) Osteocytes (OC) and osteoblasts (OB) are observed with increased magnification. (d) Defect without bone bridge formation. (e) Newly formed bone tissue on the periphery of the defect (NB). (f) Limit between preexisting mature bone tissue (PB) and newly formed bone tissue (NB). HE staining.

At 4 weeks, the absence of surface bone formation was noted in 80% of the cases; none of the defects formed a bone bridge. Peripheral immature bone formation was observed in 60% of defects, with no evidence of mature bone tissue. The immature bone was characterized by disordered bundles of collagenous fibers with randomly located osteocytes. Osteoblasts were observed at the periphery. Neoformed vessels appeared in both central and peripheral zones (60%). Loose connective tissue is formed in the central zone. At 8 weeks, the bone healing parameters showed slight variations. All samples showed peripheral bone formation, and no bone bridges were formed. At 4 weeks, bone formation was immature with the same histological features. Neoformed vessels were mainly in the central zone (50%) (Figure 4).

#### 3.2.2. A Group

**Figure 5:**
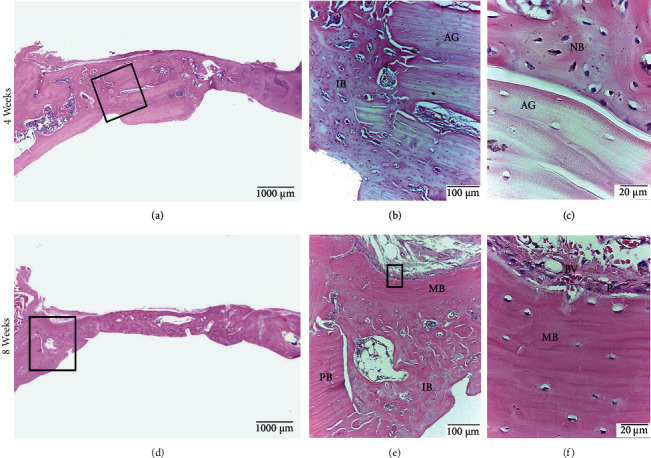
A Group. Calvarial bone defect in male Sprague Dawley rats at 4 weeks (a, b, c) and at 8 weeks (d, e, f). (a) Defect with formation of bone bridge. (b) Autograft blocks (AG) surrounded by immature bone (IB). (c) Interface between autograft block and newly formed bone (NB). (d) Defect with bone bridge formation. (e) Mature (MB) and immature bone (IB) formed in the periphery of the defect. The preexisting mature bone is also observed (PB). (f) The periosteum (P) is observed with increased magnification (P) with blood vessels (BV). HE staining.

At 4 weeks, all samples had central and peripheral bone formation; however, only 40% had a bone bridge. The immature bone surrounding the autograft blocks was compact and trabecular. Osteocytes, osteoblasts, osteoclasts, and bone canals were observed in central and peripheral zones. The periosteum was organized with abundant blood vessels. At 8 weeks, 50% of the samples showed bone formation in the central and peripheral zones, with a thick bone bridge. Immature bone tissue was located in the central and peripheral zones, and mature bone formation was mainly on the periphery. The bone tissue formed in the defect was primarily compact, and the autograft blocks could still be observed (Figure 5).

#### 3.2.3. ACS Group

**Figure 6:**
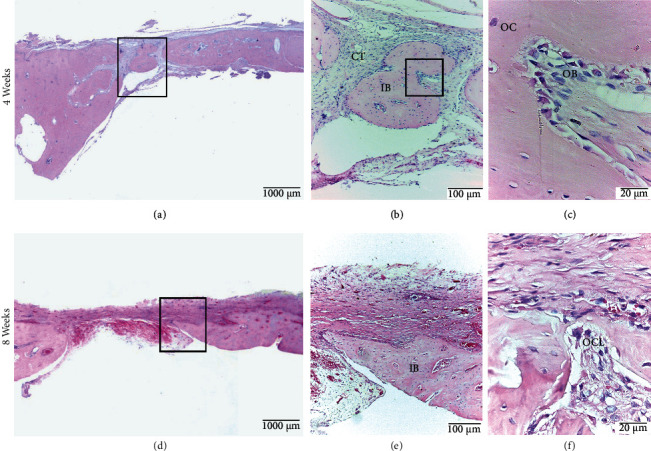
ACS Group. Calvarial bone defect in male Sprague Dawley rats at 4 weeks (a, b, c) and at 8 weeks (d, e, f). (a) Central and peripheral bone formation without bone bridge. (b) Immature bone (IB) formed at the center of the defect surrounded by connective tissue (CT). (c) Presence of osteoblasts (OB) and osteocytes (OC). (d) Defect without bone bridge formation. (e) Immature compact bone tissue on the periphery of the defect. (f) Osteoclast (OCL) in bone trabeculae. HE staining.

At 4 weeks, 80% of the samples presented with central and peripheral bone formation; however, no sample showed a bone bridge. In most cases, bone formation is immature; however, on some occasions, mature bone was observed on the periphery of the defect. Overall, 40% of the samples presented with bone trabeculae in the central and peripheral zones. Osteoblasts, osteocytes, and bone canals were observed at the periphery. Neoforming vessels appeared in both the central and lateral zones. In the 8-week group, bone formation was only observed on the periphery of the defect, and none showed bone bridge formation. The bone formation contained immature and mature bone tissue, and only 20% had bone trabeculae in the peripheral zone. The histological features of bone formation and the location of blood vessels in the defect were similar to those observed at 4 weeks. Osteoclasts were observed in the lateral area of the defect (Figure 6).

#### 3.2.4. B-TCP Group

**Figure 7:**
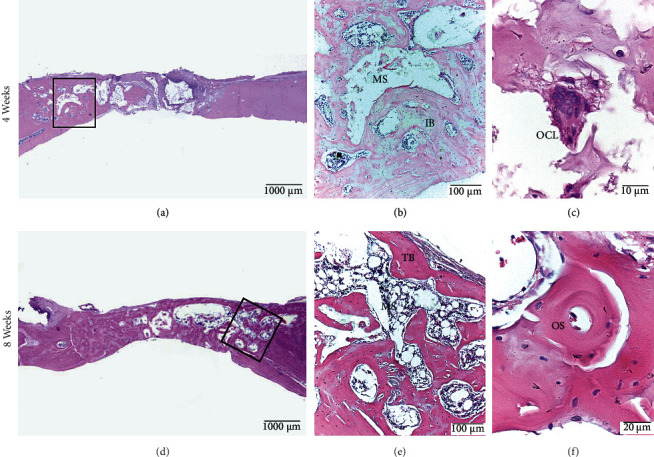
B-TCP Group. Calvarial bone defect in male Sprague Dawley rats at 4 weeks (a, b, c) and at 8 weeks (d, e, f). (a) Central and peripheral bone tissue is observed in the defect, without bone bridge formation. (b) Immature bone (IB) apposition with wide medullary spaces (MS). (c) Osteoclast (OCL) is noted with increased magnification. (d) Defect with formation of bone bridge. (e) Bone trabeculae (TB) with lamellae still disordered and wide medullary spaces (MS). (f) Osteon (OS). HE staining.

At 4 weeks, 60% of the samples had peripheral and central bone formation, and 40% of the cases formed a bone bridge. Overall, 60% of the immature bone was located in the central zone, and it was characterized by trabeculae with disorganized bone lamellae and wide medullary spaces. Neoformed vessels were mainly located in the central zone of the defect. At 8 weeks, bone formation was similar to that observed at 4 weeks; however, 60% of the samples formed a bone bridge. Mature bone was observed in the central zone of the defect. In both groups, 60% of the samples presented with central and peripheral osteoclasts, and no graft material particles were observed (Figure 7).

#### 3.2.5. B Group

**Figure 8:**
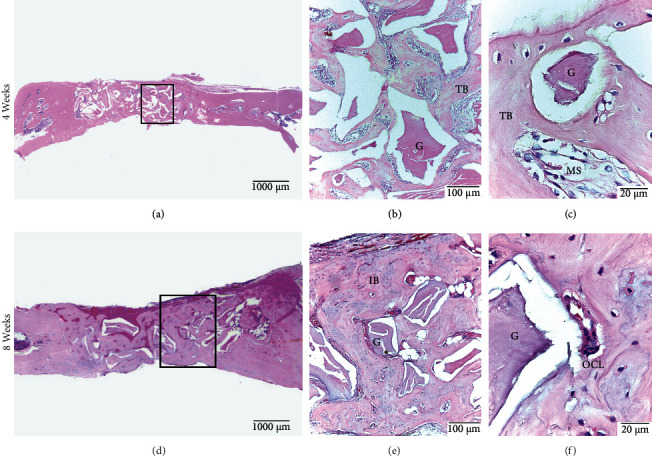
B Group. Calvarial bone defect in male Sprague Dawley rats at 4 weeks (a, b, c) and at 8 weeks (d, e, f). (a) Defect with bone bridge formation. (b) Angulated bone trabeculae (TB) surrounding particles of graft material (G). (c) Trabecular bone tissue (TB) with particles of grafted material (G) and wide medullary spaces (MS). (d) Defect with bone bridge formation with greater bone density. (e) Immature compact bone tissue (IB) surrounding particles of graft material (G). (f) Osteoclast (OCL) is noted with increased magnification. HE staining.

At 4 weeks, peripheral bone formation was observed in 60% of the cases; in the remaining 40%, bone formation was central and peripheral. Overall, 40% of the samples formed thick bone bridges. The bone was mainly immature trabeculae with wide medullary spaces, and some zones of mature bone were observed on the periphery. Graft material was noted in all samples, which were surrounded by angulated bone trabeculae as their spatial arrangement was related to the shape of the graft. Neoforming vessels appeared in both the central and peripheral zones. However, at 8 weeks, the peripheral bone formation decreased to 40%, the central and peripheral bone formation increased to 60%, and the thick bone bridge formation decreased to 20%. The bone tissue formed was similar to that in the 4-week group, but with a greater presence of mature bone tissue on the periphery. The presence of graft material and the characteristics of the trabeculae were similar to those in the 4-week group. Osteoclasts were also observed in the central and peripheral zones (Figure 8).

#### 3.2.6. HA Group

**Figure 9:**
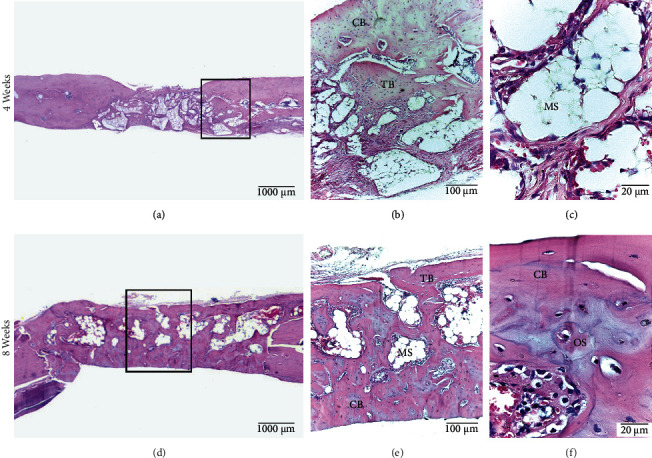
HA Group. Calvarial bone defect in male Sprague Dawley rats at 4 weeks (a, b, c) and at 8 weeks (d, e, f). (a) Defect without bone bridge formation. (b) Immature compact bone (CB) is observed on the surface and immature trabecular bone (TB) in the deepest zone. (c) Medullary spaces with adipose tissue (MS). (d) Defect with formation of bone bridge. (e) Greater presence of immature compact (CB) and trabecular bone tissue (TB) with smaller medullary spaces (MS). (f) Immature compact bone tissue (CB) with the presence of an osteon (OS) in the process of formation. HE staining.

At 4 weeks, 20% of all samples presented central and peripheral bone formation, and these cases formed a thick bone bridge. The immature bone was compact and trabecular in shape. Osteocytes, osteoblasts, osteoclasts, bone canals, and neoformed vessels were observed in both peripheral and central zones. At 8 weeks, 40% of the samples showed central and peripheral bone formation, with a thick bone bridge. The bone characteristics were similar to those observed at four weeks; however, there was a greater presence of bone tissue in the defect. Neoforming vessels appeared mainly in the central zone. In both groups, wide medullary spaces and abundant adipose tissue were noted. No graft material was observed (Figure 9).

### 3.3. Scores on the Bone Healing Scale

**Figure 10:**
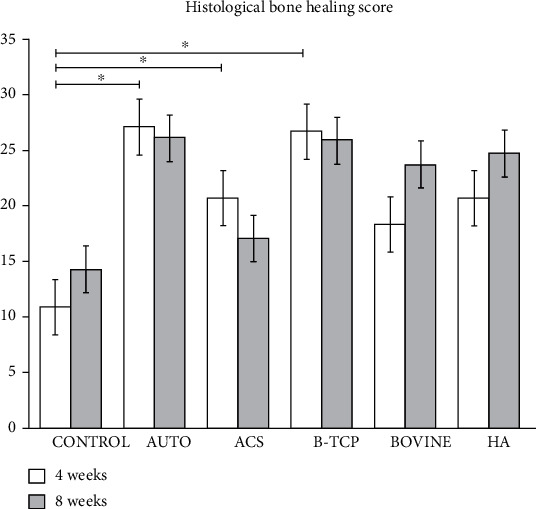
Representation of the average bone healing score (vertical axis) of the control and study groups (horizontal axis) at 4 and 8 weeks. The groups marked with ∗ have significant statistical differences (*p* < 0.05).

The average bone healing scores at 4 and 8 weeks are shown in Figure 10. At 4 and 8 weeks, the highest bone healing score was observed in A group, and the C group showed significant differences between 4 and 8 weeks (*p* = 0.032). Among the 4-week groups, the C group showed significant statistical differences from the A (*p* = 0.001), ACS (*p* = 0.017), and B-TCP (*p* = 0.005) groups. No significant differences were observed between the 8-week groups.

## 4. Discussion

The role of rhBMP-2 in bone formation has been demonstrated in the past [3, 11]. The use of materials such as HA, *β*-TCP, bovine, and autogenous particles is well known, and current clinical applications and stable results and their use in this research is based on the fact that they are frequently used in oral and maxillofacial surgery.

Although there are studies with animals that use biomaterials with rhBMP-2 [27, 28], the singularity of the animal species, anatomy of the defect, sizes of the defects, differences in the measured parameters, acquisition of data, and time at which the final response was assessed after implantation play an important role in the final outcome of the use of rhBMP-2 [28].

rhBMP-2 shows activity in osteoblast induction under certain conditions; however, rhBMP-2 can also promote osteoclast formation, resulting in resorption of the neoformed bone tissue. This occurs because of the effect of BMP on osteoclast genesis through the activation and signaling of RANKL-RANK [29–31].

Group C had the lowest bone-healing scores at 4 and 8 weeks. At 4 weeks, this group showed a significant difference from the ACS, A, and B-TCP groups; in the C group, the score increased significantly between weeks 4 and 8, but no bone bridge was observed. This confirms that the 5-mm bone defect in the rat calvaria is a critical-size defect. These results are comparable to the critical size defects of 4–8 mm reported in the literature [31–35].

Autografts are considered the gold standard treatment because of their osteoconductive, osteoinductive, and osteogenic properties. However, the donor site has complication rates between 8.6 and 20.6% [36, 37], which is the main problem when using an autograft as the sole treatment. Usually, in comparative studies, the best results are observed in autograft groups [38, 39]. In the 4-week analysis, our results showed that the autograft particles were distinguishable from the newly formed bone, and the use of this particle as the center for bone formation was also noted. At 8 weeks, however, the autograft particles were less visible because of osteoclastic degradation of the graft.

An absorbable collagen sponge (ACS) is frequently used as a scaffold for bone regeneration using rhBMP-2. However, this material has some limitations, such as rapid initial release and impossibility of long-term controlled release [15, 40]. In addition, ACS shows unpredictable biodegradation and no mechanical resistance, which makes it difficult to use in larger bone defects [16]. Factors that can modify the incorporation of rhBMP-2 into ACS include the density of ACS, sterilization method, cross-linking (formaldehyde treatment and physical methods), exposure time, concentration of rhBMP-2, pH, and type of rh-BMP-2 [14, 41].

In addition, Friess et al. [14] measured the incorporation of rhBMP-2 using ACS and showed a higher initial value with an average mean presence at the site for 81 h. The score on the bone healing scale decreased between 4 and 8 weeks (with no statistically significant differences) and could be related to the lower presence of rhBMP-2 in the long term and the use of a weak scaffold with biodegradation. *β*-TCP is a synthetic alloplastic material with osteoconductive properties [42–44]. It is a biocompatible material and can maintain its volume because of its lower resorption rate [45].

Some authors have suggested that bone formation using *β*-TCP is significantly higher with rhBMP-2 [31, 45]. However, another study showed that the addition of rhBMP-2 induces lower bone formation [43–45], possibly because using *β*-TCP with rhBMP-2 reduces the formation of the extracellular matrix, results in an increase in osteoclasts, and causes an increase in MMP-9, which suggests a rapid remodeling process [44]. In this study, the bone healing score of the *β*-TCP group was second to that of the A group; at 8 weeks, it decreased slightly compared with that at 4 weeks.

Ideally, in resorption, the grafted material is degraded synchronously with bone formation [45, 46]. Macro/microporosity is one of the main factors that affect the resorption of *β*-TCP because it allows the growth of blood vessels and osteocytic dendrites through the graft [47]. The reduced macro/micropore size can decrease bone substitution and regeneration [48]. In the *β*-TCP group, the presence of osteoclasts and degradation of the matrix in both the central and peripheral zones were noted at 4 and 8 weeks, which is consistent with the results of previous studies.

Bovine grafts are biocompatible materials frequently used in oral surgery [49]. Dehydration, sterilization, and lyophilization treatments are performed for human use, as the main risk is potential prion transmission [38]. Jung et al. [50] stated that a bovine graft in combination with rhBMP-2 increases the contact between the graft and bone tissue in humans, showing that the particle size could be an important variable [51, 52] and that the bovine particles were 100 times larger than HA/*β*-TCP [53].

In this study, the bone healing score increased from 4 to 8 weeks, which was shown to be statistically significant. Mokbel et al. [34] showed that the use of a bovine graft with rhBMP-2 resulted in greater bone formation than the autograft and allograft at 8 weeks. Nevertheless, some researchers have shown that bone formation from autografts, allografts, and bovine grafts are similar [39], while other studies have reported variable results in relation to bone formation with the bovine graft [38, 54]. We observed bone formation with peripheral and central osteoclasts at 8 weeks in the B group. This observation is consistent with the study by Issa et al., where a greater number of TRAP-positive multinucleated osteoclasts were detected compared to the autograft and allograft groups [39].

The presence of grafted materials has also been described in literature [34, 39, 50]. After three months, there was a reduction in the grafted material, and at six months, the grafted material was integrated into the bone tissue [38]. HA has a chemical composition similar to that of mineralized bone; therefore, it is biocompatible and osteoconductive [55]. However, its main disadvantages are its low resorption rate and low solubility due to the low level of carbon [56]. HA particles were present even at 6 and 8 months [38, 46, 50]. Compared with *β*-TCP, HA particles showed a lower amount of degradation [46]. It has been suggested that HA or HA/*β*-TCP improves bone regeneration by adding rhBMP-2 [50, 57]. In this study, we did not observe any statistically significant differences in bone formation between 4 and 8 weeks in the HA group. Moreover, the result in HA group was comparable to B and *β*-TCP groups, suggesting a similar effect on bone formation [58], indicating that the ability of rhBMP-2 to act as an osteoconductive factor showed no differences between the grafted materials.

## 5. Conclusions

A rat calvarial 5-mm bone defect is a critical-size defect that requires the use of biomaterials to heal at 4 and 8 weeks. The histological characteristics of the B-TCP group were similar to those of bone regeneration in the autograft group. The use of *β*-TCP with rhBMP-2 is an alternative therapy, which may preclude the morbidity associated with autograft harvest as well as its associated complications, and lower costs due to extended hospitalization.

## Figures and Tables

**Table 1:** Histological evaluation record.

Histological score
*1. Bone formation*
0 – Absent
1 – Present at the periphery
2 – Present centrally
3 – Present centrally and at the periphery
*2. Bone formation*
0 – Absent
1 – Present at the surface of the graft
2 – Present in the depth of the graft
*3. Vascularization of the graft*
0 – Absent
1 – Present at the surface of the graft
2 – Present in the depth of the graft
*4. Osteoblasts*
0 – Absent
1 – Present at the periphery
2 – Present centrally
3 – Present centrally and at the periphery
*5. Osteocytes*
0 – Absent
1 – Present at the periphery
2 – Present centrally
3 – Present centrally and at the periphery
*6. Osteoclasts*
0-absent
1 – Present at the periphery
2 – Present centrally
3 – Present centrally and at the periphery
*7. Immature bone*
0 – Present centrally
1 – Present at the periphery
2 – Absent
*8. Mature bone*
0 – Absent
1 – Present at the periphery
2 – Present centrally
3 – Present at the periphery and centrally
*9. Osteoclastic degradation of the scaffold*
0 – Absent
1 – Present at the periphery
2 – Present centrally
3 – Present centrally and at the periphery
*10. Scaffold replacement with mature bone*
0 – Absent
1 – Present at the periphery
2 – Present centrally
3 – Present at the periphery and centrally
*11. Bone bridge*
0 – Absent
1 – Narrow
2 – Thick
*12. Bone trabeculae*
0 – Absent
1 – Present at the periphery
2 – Present centrally
3 – Present at the periphery and centrally
*13. Haversian canals*
0 – Absent
1 – Present at the periphery
2 – Present centrally
3 – Present at the periphery and centrally
*14. Inflammation*
0 – Present
1 – Absent
*15. Granulation tissue*
0 – Present
1 – Absent
*16. Neoformation of blood vessels*
0 – Absent
1 – Present at the periphery
2 – Present centrally
3 – Present centrally and at the periphery
*17. Bone tissue*
0 – Absent
1 – Present at the periphery
2 – Present centrally
3 – Present at the periphery and centrally

**Table 2:** Characterization of experimental groups using the healing score (histological parameters) of male Sprague Dawley rat calvarial critical size defects at 4 and 8 weeks after surgery.

Histological bone healing parameters		Group frequency (%)											
		C		A		ACS		B-TCP		B		HA	
		4 W	8 W	4 W	8 W	4 W	8 W	4 W	8 W	4 W	8 W	4 W	8 W
*Surface bone formation*	Absent	80	0	0	0	0	0	0	0	0	0	0	0
	Peripheral	20	100	0	40	20	100	40	40	60	40	80	60
	Central	0	0	0	0	0	0	0	0	0	0	0	0
	Central and peripheral	0	0	100	60	80	0	60	60	40	60	20	40
*Bone formation in the depth of the graft*	Absent	60	0	0	0	0	0	0	0	0	0	20	0
	Surface	20	80	20	100	0	0	0	0	40	0	40	0
	Profound	20	20	80	0	100	100	100	100	60	100	40	100
*Graft vascularization*	Absent	0	0	0	0	0	0	0	0	20	0	0	0
	Surface of the graft	20	80	0	0	0	0	0	40	20	0	40	0
	Depth of the graft	80	20	100	100	100	100	100	60	60	100	60	100
*Osteoblasts*	Absent	40	0	0	0	0	0	0	0	0	0	0	0
	Peripheral	60	80	0	40	20	100	20	40	60	40	80	60
	Central	0	0	0	0	0	0	0	0	0	0	0	0
	Central and peripheral	0	20	100	60	80	0	80	60	40	60	20	40
*Osteocytes*	Absent	40	0	0	0	0	0	0	0	0	0	0	0
	Peripheral	60	80	0	40	20	100	20	40	60	40	80	60
	Central	0	0	0	0	0	0	0	0	0	0	0	0
	Central and peripheral	0	20	100	60	80	0	80	60	40	60	20	40
*Osteoclasts*	Absent	100	100	40	0	100	40	0	20	100	40	0	0
	Peripheral	0	0	20	40	0	60	40	20	0	40	0	0
	Central	0	0	0	0	0	0	0	0	0	20	20	40
	Central and peripheral	0	0	40	60	0	0	60	60	0	0	80	60
*Immature bone*	Central	0	0	100	40	80	0	60	60	40	40	20	40
	Peripheral	60	100	0	60	20	100	40	40	60	60	80	60
	Absent	40	0	0	0	0	0	0	0	0	0	0	0
*Mature bone*	Absent	100	100	80	40	80	40	40	80	80	20	100	100
	Peripheral	0	0	20	60	20	60	60	0	20	80	0	0
	Central	0	0	0	0	0	0	0	20	0	0	0	0
	Central and peripheral	0	0	0	0	0	0	0	0	0	0	0	0
*Osteoclastic degradation of scaffold*	Absent	100	100	40	0	100	40	0	20	100	40	0	0
	Peripheral	0	0	20	40	0	60	40	20	0	40	0	0
	Central	0	0	0	0	0	0	0	0	0	20	20	40
	Central and peripheral	0	0	40	60	0	0	60	60	0	0	80	60
*Scaffold replacement w/mature bone*	Absent	100	100	100	40	100	60	40	80	80	20	100	100
	Peripheral	0	0	0	60	0	40	60	0	20	80	0	0
	Central	0	0	0	0	0	0	0	20	0	0	0	0
	Central and peripheral	0	0	0	0	0	0	0	0	0	0	0	0
*Bone bridge*	Thin	100	100	60	60	100	100	60	40	60	80	80	60
	Thick	0	0	20	0	0	0	20	0	0	0	0	0
	Absent	0	0	20	40	0	0	20	60	40	20	20	40
*Bone trabeculae*	Absent	60	80	20	80	60	80	20	0	0	0	60	40
	Peripheral	40	20	60	20	0	20	0	40	60	40	20	20
	Central	0	0	20	0	0	0	20	40	20	40	20	0
	Central and peripheral	0	0	0	0	40	0	60	20	20	20	0	40
*Haversian canals*	Absent	100	100	0	0	40	40	0	20	80	0	60	0
	Peripheral	0	0	60	40	60	60	80	40	0	100	40	80
	Central	0	0	0	0	0	0	0	0	0	0	0	0
	Central and peripheral	0	0	40	60	0	0	20	40	20	0	0	20
*Inflammation*	Present	0	0	0	0	0	0	0	0	20	0	20	20
	Absent	100	100	100	100	100	100	100	100	80	100	80	80
*Granulation tissue*	Present	0	0	0	0	0	0	20	0	20	0	20	0
	Absent	100	100	100	100	100	100	80	100	80	100	80	100
*Neoformation vessels*	Absent	0	0	0	0	0	0	0	0	0	0	0	0
	Peripheral	20	20	0	0	40	0	0	0	20	0	0	0
	Central	20	60	20	0	40	0	100	60	60	100	20	80
	Central and peripheral	60	20	80	100	20	100	0	40	20	0	80	20
*Bone tissue*	Absent	40	0	0	0	0	0	0	0	0	0	0	0
	Peripheral	60	80	0	40	20	100	40	40	60	40	80	60
	Central	0	0	0	0	0	0	0	0	0	0	0	0
	Central and peripheral	0	20	100	60	80	0	60	60	40	60	20	40

## Data Availability

All the data supporting this study are included in the article.
